# Dysregulation in Actin Cytoskeletal Organization Drives Increased Stiffness and Migratory Persistence in Polyploidal Giant Cancer Cells

**DOI:** 10.1038/s41598-018-29817-5

**Published:** 2018-08-09

**Authors:** Botai Xuan, Deepraj Ghosh, Emily M. Cheney, Elizabeth M. Clifton, Michelle R. Dawson

**Affiliations:** 1 Brown University, Department of Molecular Pharmacology, Physiology, and Biotechnology, Providence, 02912 USA; 2 Brown University, Center for Biomedical Engineering, Providence, 02912 USA; 3 Brown University, School of Engineering, Providence, 02912 USA

## Abstract

Polyploidal giant cancer cells (PGCCs) have been observed by pathologists in patient tumor samples and are especially prominent in late stage, high grade disease or after chemotherapy. However, they are often overlooked due to their apparent dormancy. Recent research has shown PGCCs to be chemoresistant and express stem-like features, traits associated with disease progression and relapse. Here, we show the preferential survival of PGCCs during Paclitaxel (PTX) treatment and used multiple particle tracking analysis to probe their unique biophysical phenotype. We show that PGCCs have higher inherent cytoplasmic and nuclear stiffness in order to withstand the mechanical stress associated with their increased size and the chemical stress from PTX treatment. Inhibitor studies show the involvement of a dysregulated RhoA-Rock1 pathway and overall actin cytoskeletal network as the underlying mechanism for the altered biophysical phenotype of PGCCs. Furthermore, PGCCs exhibit a slow but persistent migratory phenotype, a trait commonly associated with metastatic dissemination and invasiveness. This work demonstrates the clinical relevance and the need to study this subpopulation, in order to devise therapeutic strategies to combat disease relapse. By highlighting the unique biophysical phenotype of PGCCs, we hope to provide unique avenues for therapeutic targeting of these cells in disease treatment.

## Introduction

Breast cancer is one of the leading causes of cancer related mortality in women with more than 1.3 million cases diagnosed annually and 450 thousand deaths per year worldwide^1^. In part to earlier detection and combination chemotherapy regimens, endocrine, and HER2-targeting therapies the rate of breast cancer mortality has fallen in the last two decades^2^; however, there has been more limited progress in incorporating adjuvant therapies in the treatment of triple negative breast cancer (TNBC)^3^. TNBC represents a heterogeneous group of highly aggressive tumors that lack hormone and HER2/ERBB2 receptors, which are critical in targeting cancer cells. While chemotherapeutic agents like Taxol are first-line treatment for TNBC, tumor reoccurrence after chemotherapy is a major problem and is often associated with metastatic and drug-resistant cancer^4^. TNBC, which is more common in young and African American women, has grim clinical outcomes. New treatment options are urgently needed. Increased understanding of how breast cancer stem cells survive chemotherapy and go on to form drug-resistant tumors is critical in developing better strategies for treating TNBC.

Cancer cell dormancy poses significant challenges in cancer treatment. Current chemotherapeutic regiments primarily target rapidly dividing cells. As such, cancer cells that undergo transient quiescence are capable of escaping treatment and cause disease relapse after exiting their quiescent state^5,6^. One subpopulation that is thought to utilize quiescence and amitotic division to escape treatment is polyploidal giant cancer cells (PGCCs). Histopathological analysis of human tumor tissue has shown the existence of these large aberrant multinucleated cancer cells^7^. PGCCs exist in pre-malignant tissues but are especially prominent in high grade, late stage disease or after chemotherapy^8–10^, which suggests a link between these abnormal cells and the potential for tumor recurrence. Furthermore, *in vitro* studies have also shown that PGCCs found in MDA-MB-231 and MCF7 breast cancer cell lines display a stem like phenotype characterized by spheroid formation, asymmetric division by amitotic budding, and the ability to differentiate along multiple lineages^11,12^. In addition, it is believed that the extra-chromosomal content of PGCCs confer resistance to DNA damage and give rise to complex tumor cell karyotypes, further increasing genetic diversity in cancer^13^. Given the clinical challenges these PGCCs present, it is imperative to study this subpopulation to devise therapeutic strategies to mitigate their deleterious effects.

Despite the multifaceted ability of PGCCs to contribute to drug resistance and relapse, no in-depth biophysical analysis of these cells has been conducted. To fully probe the behavior and tumorigenic properties of these PGCCs, it is critical to understand their biophysical properties. Many of the hallmarks associated with cancer, including unlimited replicative potential, apoptotic evasion, tissue invasion and metastasis, can be linked to abnormal cytoskeletal or matrix mechanics – important biophysical parameters^14^. Moreover, targeting the biophysical characteristics that allow PGCCs to survive both the mechanical stress associated with their increased size and chemical stress induced by chemotherapy could provide a novel avenue of therapeutic treatment that can be adjuvant to mainline treatments in the clinical setting.

In this study, we sought to probe the biophysical phenotype and associated underlying mechanisms of MDA-MB-231 PGCCs. In addition, we also examined their unique morphological and migratory phenotype. We focused on MDA-MB-231, a triple negative breast cancer line due to their highly invasive nature and high rates of recurrence, aspects we are interested in within the context of PGCCs. MDA-MB-231 PGCCs exhibit increased stiffness in both cytoplasmic and nuclear mechanics compared to normal non-polyploidal (non-PGCC) MDA-MB-231 cells. PGCCs had dramatic differences in the organization of actin stress fibers, including longer and thicker stress fiber bundles. The increased cytoskeletal stiffness and nuclear structure was largely regulated through the RhoA-ROCK1 pathway and actin cytoskeletal dynamics, which are essential to their biophysical phenotype. Furthermore, PGCCs demonstrated an altered migratory pattern. While PGCCs move more slowly, their motion is more persistent, allowing them to move longer distances over time. The directional migration of PGCCs was associated with a highly deformable nuclear structure, which is characterized by a unique softening of the nucleus during periods of migration. In sum, PGCCs display a distinctively altered and dysregulated biophysical phenotype, which is governed by alterations in actin cytoskeletal organization.

## Results

### Subpopulation of breast cancer cells exhibits aberrant nuclear morphology and chemoresistance

**Figure 1:**
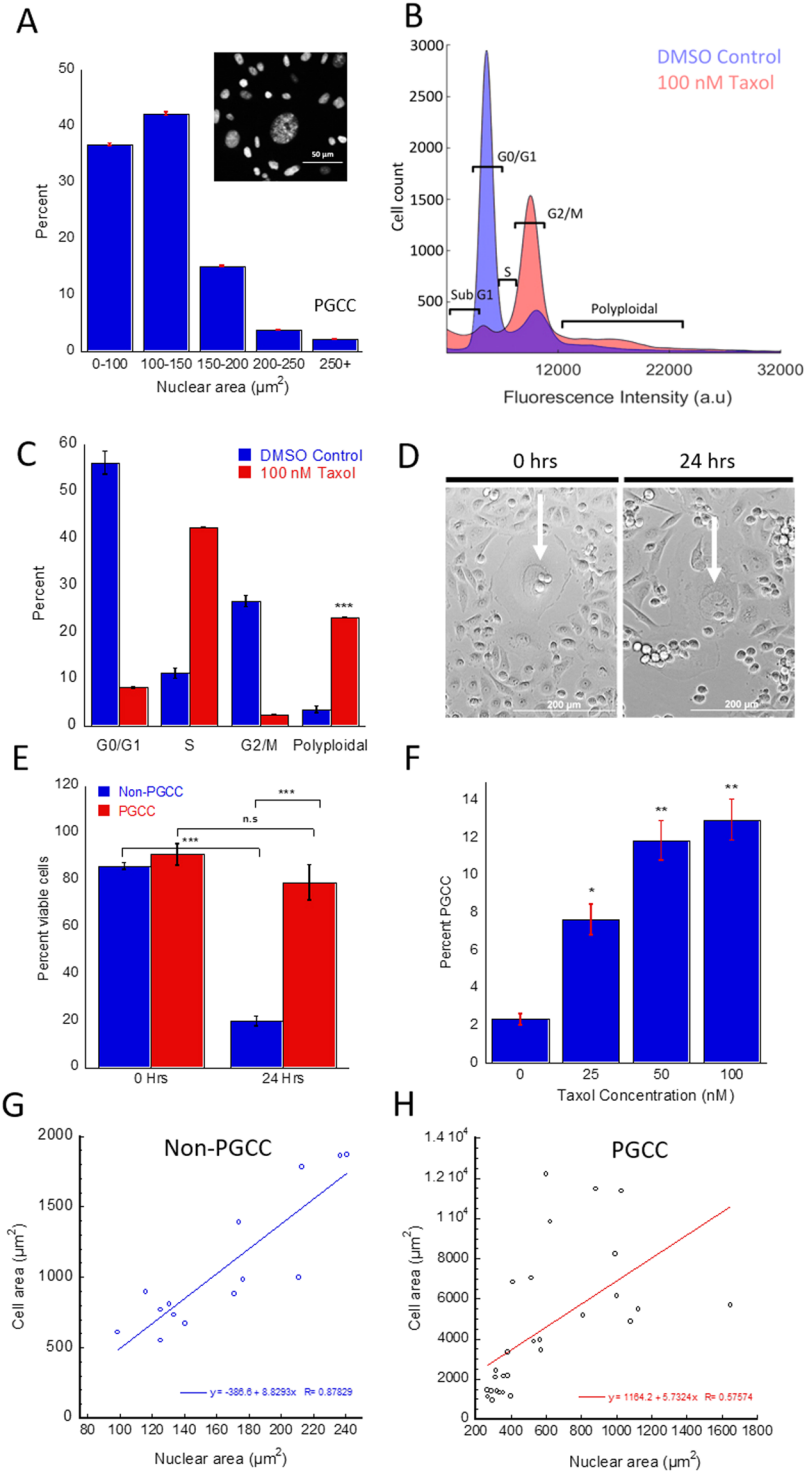
Characterizing PGCC occurrence and PTX resistance in MDA-MB-231 cancer cells. (**A**) Nuclear area distribution of MDA-MB-231 cells stained with DAPI (n = 5, 5000 + cells). (**B**) Cell cycle analysis via flow cytometry of DMSO and 48-hr 100 nM PTX treated cells show different stages of cell cycle and polyploidy. (**C**) Quantification of cell cycle analysis for DMSO and 100 nM PTX treated cells reveal increased PGCC occurrence. (**D**) Time lapse images of MDA cells before and after 24-hr treatment with 100 nM PTX (PGCCs indicated by white arrow). (**E**) Quantification of time lapse cell survival (500 + cells) show PGCC resistance to treatment. (**F**) PGCC incidence rate rises with increasing doses of 48-hr PTX treatment (n = 4). (**G**) Nuclear and cell area correlation of non-PGCCs and (**H**) PGCCs (40 + cells) show decreased correlation in the PGCC subpopulation. Cell cycle analysis and time lapse experiments were performed in triplicate. Results are reported as the mean ± SEM. Significance is indicated as *p < *0.05*, **p < *0.01*, ***p < *0.001*.

Despite the relevance of PGCCs in the context of chemoresistance and disease relapse, they remain poorly characterized. PGCCs have previously been observed in cancer cell lines as a small but significant subpopulation that is amplified upon Paclitaxel (PTX) treatment^11,12,15^. We confirmed and quantified this observation in MDA-MB-231 cells through immunostaining and cell cycle analysis via flow cytometry. Cells containing more than 2.5 times (250 µm^2^+ for MDA-MB-231 cells) normal DNA content (as characterized by nuclear area or PI fluorescence) were found to comprise between 2 to 5 percent of total cell population (Figs 1A–C and S1E). Our polyploidal population contained a wide range of chromosomal content in excess of 4N, suggesting multiple mechanisms contribute to their formation, leading to levels of excess DNA duplication (4N, 8N, 16N+). To ensure the increase in nuclear size is indicative of increased DNA content and not differences in binding affinity of DAPI dye to DNA, we analyzed the fluorescence intensity as a function of nuclear area and found no statistical differences between intensity per area for PGCCs and non-PGCCs (p = 0.182) (Fig. S1). Morphological quantification of cell area revealed a strong correlation between nuclear area and cell size (Fig. S1A). However, this trend was significantly weaker for PGCCs, suggesting a dysregulation in mechanisms that govern the ratio of nuclear to cytosplasmic volume (Fig. 1G,H). The change of cell area as a function of nuclear area was lower for PGCCs than non-PGCCs, as evidenced by the reduced slope for the correlation. The reduced slope shows PGCC cell area did not increase at the same rate with nuclear area, which could indicate a shorter growth phase in the cell cycle (less time spent in G1/G2). Alternatively, an increase in cell thickness may be responsible for this difference. Volume analysis show a 13-fold increase in volume for PGCCs compared to non-PGCCs (p = 0.0006), but only a 6-fold increase in average cell area (p = 0.0009) (Fig. S1B,C), pointing towards increased thickness as a factor in the lower rate of correlation, at least in part. Upon treatment with PTX, the PGCC subpopulation was significantly increased in a concentration dependent manner (Fig. 1C,F), ranging from a 3-fold increase at 25 nM PTX (p = 0.032) up to a 10-fold increase at 100 nM PTX treatment for 48 hours (p = 0.007). We hypothesized that this increase in PGCC population was due to their abnormal phenotype, which conferred a survival advantage to these cells during PTX treatment. To confirm our hypothesis, we captured 24 hour time-lapse videos (Fig. 1D) of MDA-MB-231 cells treated with 100 nM PTX and analyzed the number of apoptotic cells. Apoptotic cells were identified by rapid changes in cell shape from adherent and spread to detached and rounded, as described previously^16^. Non-PGCCs show significant cell death, while PGCCs were able to survive the 24-hour treatment (Fig. 1E). To determine how PGCCs withstand the mechanical stress associated with their increased size and the chemical stress from PTX treatment, a single cell biophysical approach was used to investigate fundamental differences in PGCCs and non-PGCCs.

### PGCCs demonstrate altered cytoskeletal mechanics

**Figure 2:**
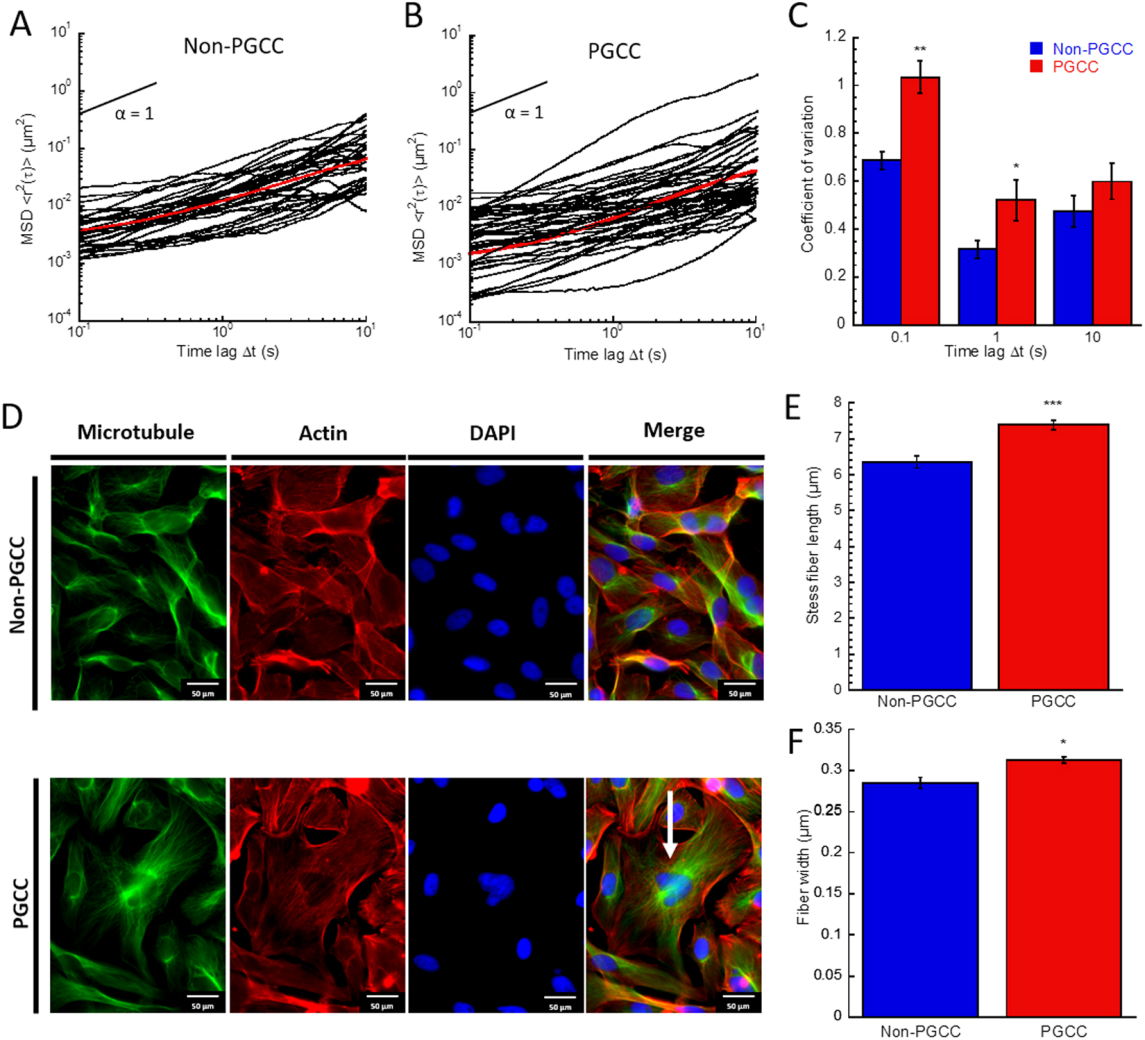
Altered cytoskeletal organization drive increased stiffness in PGCCs. (**A**) Ensemble averaged MSDs derived from particle motion embedded within cell cytoplasm of non-PGCCs and (**B**) PGCCs show decreased particle motion and increased stiffness in PGCCs (over 100 traces). **(C)** Coefficient of variation analysis for ensemble MSDs of non-PGCC and PGCCs reveal increased heterogeneity in PGCCs. **(D)** Fluorescent images of non-PGCC and PGCCs (white arrow) stained with Phalloidin (F-actin, *red*), Anti-α-tubulin (Microtubule, *green*), and DAPI (Nucleus, *blue*). **(E)** Actin stress fiber quantification show increased stress fiber length and **(F)** width in PGCCs compared to non-PGCCs. Results are reported as the mean ± SEM. Significance is indicated as *p < *0.05*, **p < *0.01*, ***p < *0.001*.

First, multiple particle tracking microrheology (MPT) was used to determine cell mechanical properties from the thermal motion of ballistically injected fluorescent nanoparticles^17^. This technique was used to probe the local mechanical properties in individual cells (PGCCs and non-PGCCs) based on the thermal displacements of 200-nm particles embedded in the cell cytoplasm (10–20 particles per cell). For tracking studies, PGCCs and non-PGCCs were identified based on differences in nuclear size. For each condition we report the ensemble averaged MSD <<r^2^(τ)>> of individual particle traces (n~30 cells and ~100 traces per condition). The logarithmic slope of MSD for PGCCs and non-PGCCs was similar (0.69 < α < 0.76), in the range indicating viscoelastic behavior (Fig. 2A,B). However, ensemble averaged PGCC particle traces show a 2-fold reduction in amplitude across all time lags, indicating inherent stiffness. This inherently stiffer cytoplasm would be required to withstand increased mechanical stresses associated with the increased size of PGCCs. The lower amplitude of particle traces observed in the PGCC subpopulation represent increased constraint of particles within the cytoskeletal network, as individual particle displacements are dependent on their interactions with surrounding cytoskeletal elements. Since each particle only probes the mechanical properties of their local environment, we can analyze the variation in particle MSDs to gain insight on the heterogeneity of mechanical properties. Overall heterogeneity of PGCCs was significantly higher than non-PGCCs. The coefficient of variation (standard deviation divided by the mean) of individual particle MSDs at a time lag of 0.1 seconds (Fig. 2C) in addition to ensemble averaged MSD per cell (Fig. S2C) was higher, suggesting higher heterogeneity not only at the population scale but also on the local regional level within the cell. Due to the nature of MPT, we suspected that the altered mechanical phenotype exhibited by the PGCCs as well as associated heterogeneity was tightly linked to the cytoskeletal network of the cell, as proposed by previous studies^17^. To confirm, we visualized the actin and microtubule networks of PGCCs and non-PGCCs via immunostaining (Fig. 2D). The images show increased appearance of actin stress fibers and abnormal microtubule organization. Further quantification of actin stress filaments showed a ~20% (p = 0.003) and ~12% (p = 0.04) increase in actin stress fiber length and width (Fig. 2E,F), respectively. This suggested differential organization and coverage increase of actin stress fibers contributed to localized regions and overall stiffness within the PGCCs.

### Mechanical properties of PGCC nuclei are characterized by elevated stiffness

**Figure 3:**
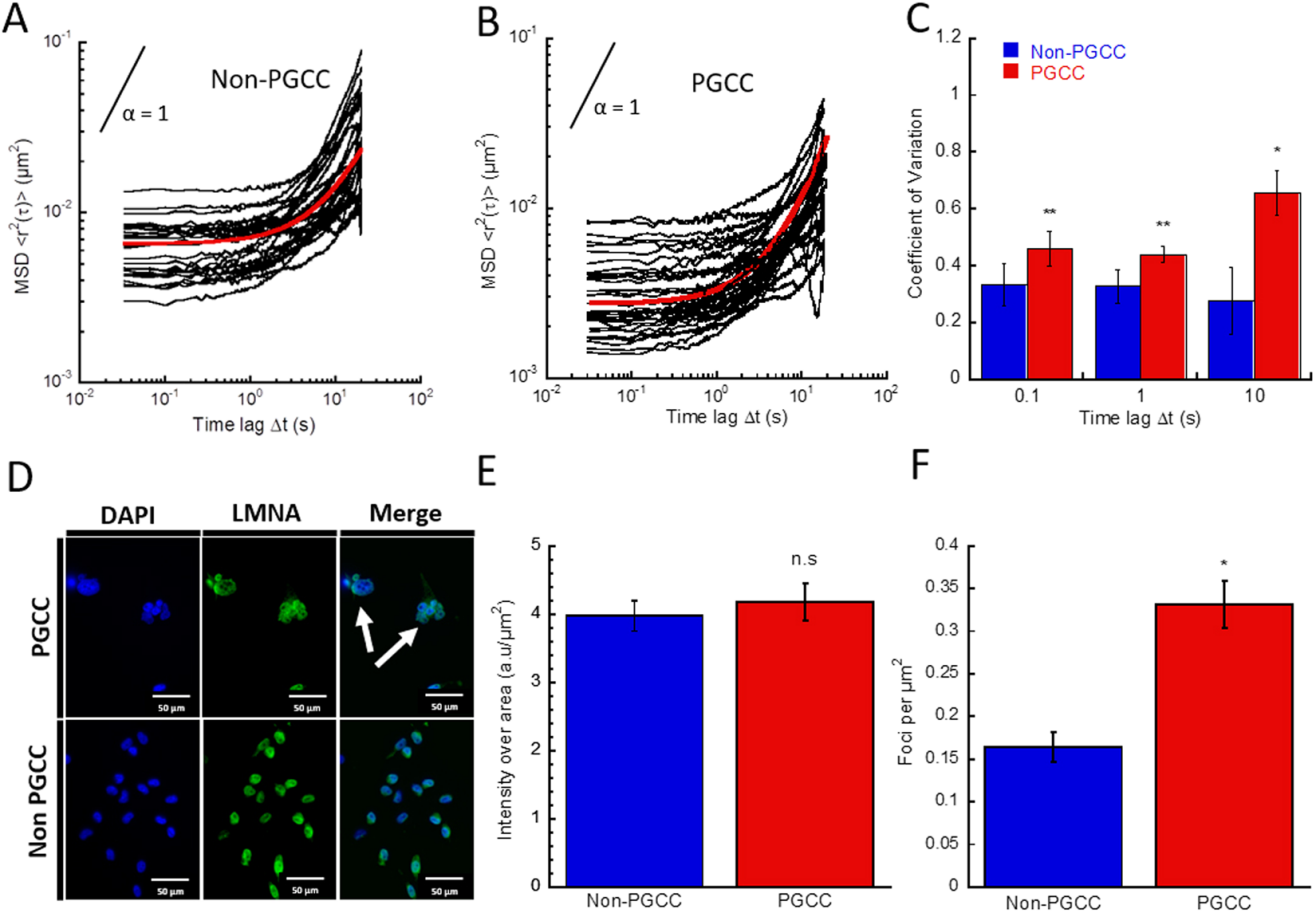
Nuclear biomechanical analysis reveal stiffer nuclei in PGCCs. Ensemble averaged MSDs derived from chromatin granule motion within nuclei of (**A**) non-PGCCs and (**B**) PGCCs show decreased particle motion and increased nuclear stiffness in PGCCs (100 + traces, 30 + cells). (**C**) Coefficient of variation analysis for ensemble MSDs of non-PGCC and PGCCs reveal increased heterogeneity in nuclear mechanics for PGCCs. (**D**) Fluorescent images of non-PGCC and PGCCs (white arrow) stained with anti-lamin A/C (nuclear lamin, *green*) and DAPI (nucleus, *blue*). (**E**) Fluorescence intensity quantification of lamin A/C expression in MDA cells reveal no significant differences between PGCCs and non-PGCCs. (**F**) Quantification of heterochromatic foci density show increased number of foci as a function of area in PGCCs. Results are reported as the mean ± SEM. Significance is indicated as *p < *0.05*, **p < *0.01*, ***p < *0.001*.

Given the close interplay between cytoskeletal and nuclear mechanics, next we wanted to investigate if the stiffer cytoplasmic mechanics of PGCCs and upregulation of F-actin bundled elements would manifest within nuclear behavior in similar fashion. Multiple particle analysis was conducted on Hoescht-stained chromatin granules within PGCC and non-PGCCs as described previously^17^. Consistent with previous studies, both PGCCs and non-PGCCs initially exhibited behavior akin to an elastic solid, represented by a MSD slope close to 0, but at longer time lags nuclear behavior transitioned to a more viscous nature, corresponding with an increasing MSD slope tending towards 1 (representing a perfectly viscous fluid). However, similar to cytoplasmic mechanics, the amplitude of PGCC nuclear particle MSDs shows a significant decrease compared to non-PGCCs (Fig. 3A,B), suggesting an inherently stiffer nuclear structure at lower time lags. Relaxation time quantifies the transition from elastic to viscous character and is defined as when the slope of the MSD reaches 0.5. Surprisingly, PGCCs demonstrated a faster transition to viscous character, with a relaxation time of 5.73 seconds compared to non-PGCCs at 6.52 seconds. This highlights differences in the dynamics of PGCC nuclear mechanics, allowing a rapid transition from an initially elastic state to a viscous one. Heterogeneity analysis of individual particle MSDs further show a greater variation in nuclear mechanical properties in PGCCs compared to non-PGCCs at for all time lags on an individual particle level (Fig. 3C), indicating uneven regions of differing stiffnesses present within the nucleus. To determine if this difference in overall nuclear stiffness is due to a dysregulation in nuclear lamina, we fluorescently stained for nuclear envelope associated protein lamin A/C (Fig. 3D) and quantified expression around the nucleus. We found that lamin A/C expression as a function of nuclear area remains unchanged between PGCCs and non-PGCCs (Fig. 3E). However, quantification of intense chromatin granules (representative of heterochromatic foci^18^) in high magnification fluorescent images show a significantly increased number of foci as a function of nuclear area (Fig. 3F), which could comprise the inherently stiff regions of the nuclei, containing highly condensed chromatin^18^.

### PGCCs migrate slowly but with more persistence

**Figure 4:**
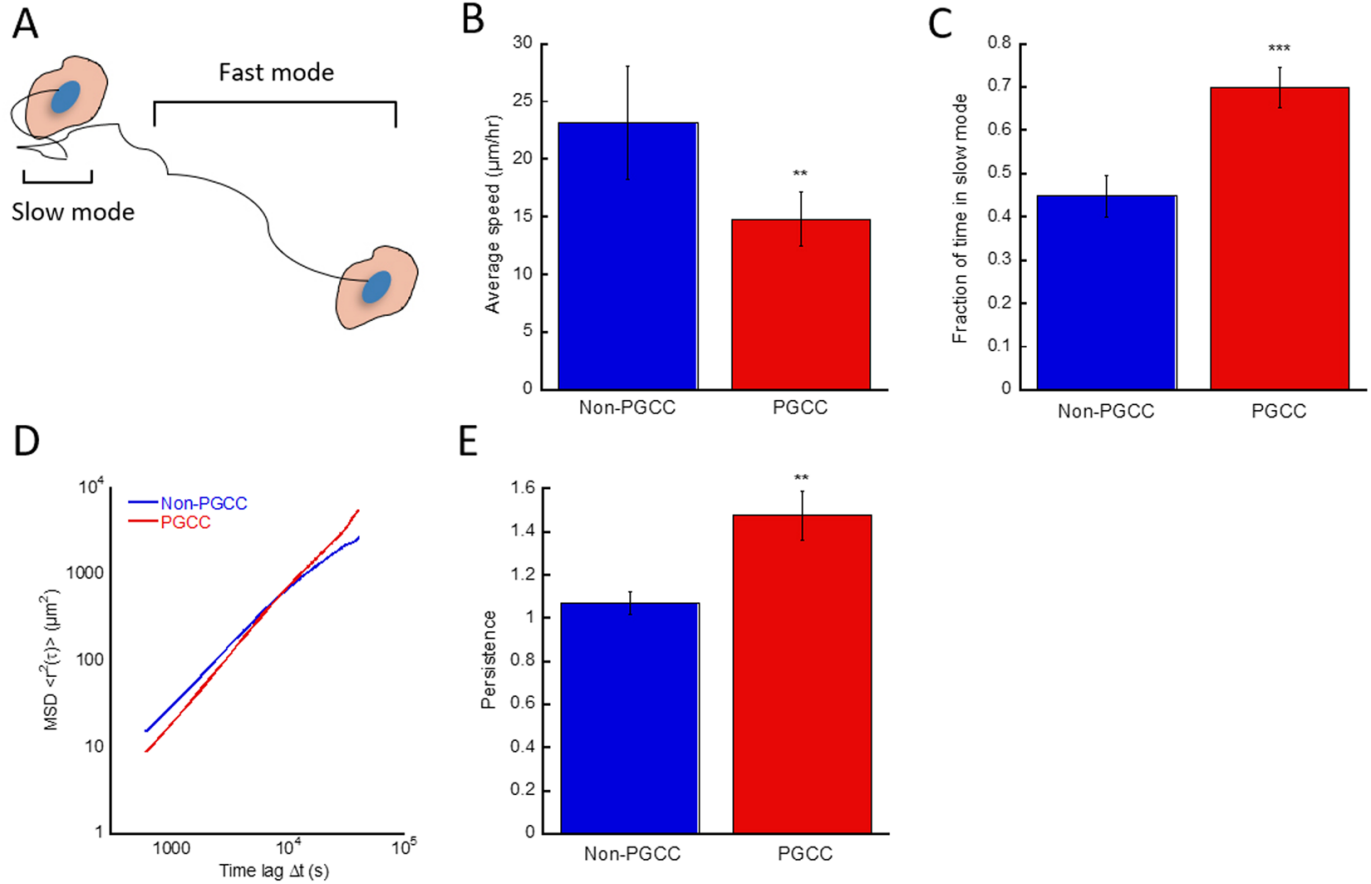
PGCCs exhibit a more persistent migratory phenotype. (**A**) Schematic depicting the two modes of movement during cell migration. The slow and fast mode cutoff is defined as movement of at least 1 average cell length per hour. (**B**) Average speed of random migration and **(C)** Fraction of time spent in the slow mode of movement for non-PGCCs and PGCCs. (**D**) Cell motion derived MSDs of PGCCs non-PGCCs during 12-hr migration assay (n = 5, 50+ cells). (**E**) Persistence analysis of non-PGCCs and PGCCs show increased directional persistence during migration. Significance is indicated as *p < *0.05*, **p < *0.01*, ***p < *0.001*.

Previous studies have shown cell mechanics and migration is directly correlated^19,20^. To examine the migration of PGCCs, MDA-MB-231 cells were seeded on collagen coated 24 well plates to evaluate their motility. At lower time lags, non-PGCCs exhibited ~57% higher speeds of migration compared to PGCCs (p = 0.0056) (Fig. 4B). This difference in average speed can primarily be attributed to an incongruity of time spent in the fast versus slow modes of migration (Fig. 4A) (cutoff between slow and fast modes defined by movement of at least 1 normal cell length within 1 hour) of these MDA-MB-231 cells. On average, PGCCs spent 56% more time in the slow mode of movement (p = 0.0003) as opposed to non-PGCCs (Fig. 4C). However, despite the slower cell velocity, the directional persistence of PGCCs was significantly higher. This manifested in the MSD traces of cell tracks during the longer time lags of 150 minutes or more, at which point the PGCCs surprisingly covered a larger net distance than non-PGCCs (Fig. 4D). This observation is further reinforced by persistence analysis (determined by the logarithmic slope of the MSD, with 1 being a randomly moving cell and 2 being a cell moving in a straight line) of PGCC and non-PGCC motility, where PGCCs on average were ~35% more persistent in their migration (p = 0.0042) (Fig. 4E). Increased persistence has been previously associated with cancer invasiveness^21,22^, leading to metastatic dissemination into surrounding tissue. Overall, PGCCs exhibit a slow but steady migratory phenotype, dictated by their abnormal cytoskeletal organization and large size.

### PGCCs nuclei deform more readily in response to scratch

**Figure 5:**
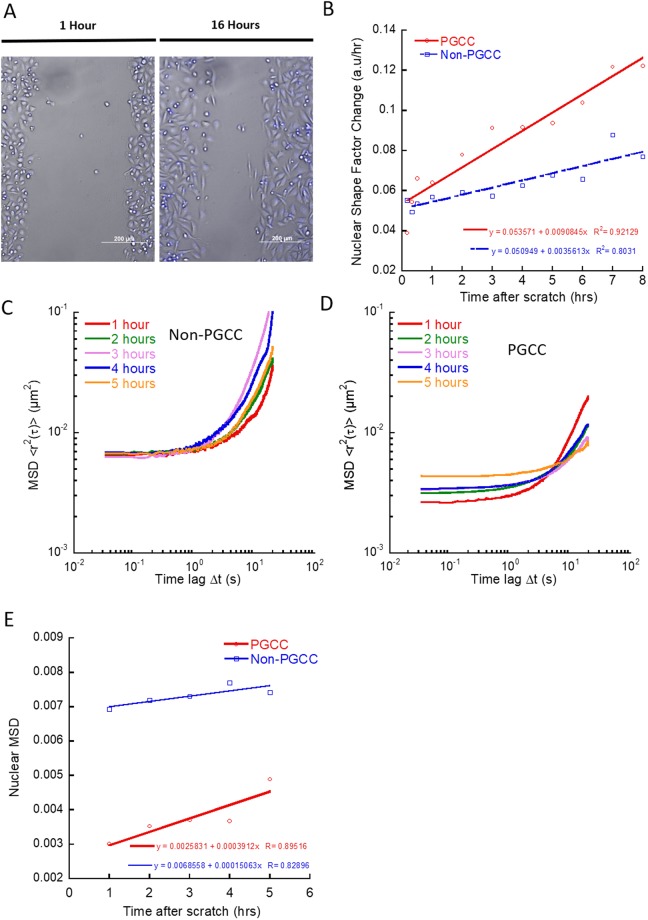
PGCCs have more deformable nuclei and alter nuclear mechanics during migration. (**A**) Time lapse images of confluent monolayer with scratch closing over the course of 16 hours. (**B**) Rate of nuclear deformation measured as the change in nuclear shape factor from 0 to 8 hours after initial scratch (30 + cells). (**C**) Total averaged ensemble MSDs of tracked chromatin granules in non-PGCCs and (**D**) PGCCs hours after scratch (10 + cells). (**E**) MSD at 1 second time lag of non-PGCC and PGCCs hours after scratch show increased softening of PGCC nuclei compared to non-PGCCs.

Nuclear deformation during cell migration is an essential part of the migratory process, not only for nuclear relocation during migration but also during regions of confinement^23^. To investigate the dynamics of nuclear deformation in PGCCs, we looked at their nuclear deformation during induced directional movement via scratch assay (Fig. 5A). True to their lower nuclear relaxation time, PGCCs were able to alter their nuclear shape factor at a faster rate than non-PGCCs, with increased rate of nuclear shape factor change (shape factor of 1 defined as circular and 0 as a line) measured at 1 to 8 hours after scratch (Fig. 5B). To further probe the behavior of the PGCC nuclei during directed migration, we examined the nuclear mechanics of PGCCs at each hour after the initial scratch was made. Averaged ensemble MSD of all cells taken at short time scales (time lag < 1 second) revealed a softening of PGCC but not non-PGCC nuclei in response to induced migration (Fig. 5C,D) as marked by increasing amplitudes in MSDs. However, PGCC relaxation times were also increased in response to the scratch, pointing towards a reorganization of chromatin structure that allowed PGCCs to soften their nuclei but at the same time limited the dynamics of their relaxation. Taken together, these results suggest PGCCs alter their chromatin organization in order to facilitate periods of rapid migration, contributing to their invasiveness.

### RhoA-ROCK1 and actin cytoskeleton regulates cytoplasmic and nuclear mechanics

**Figure 6:**
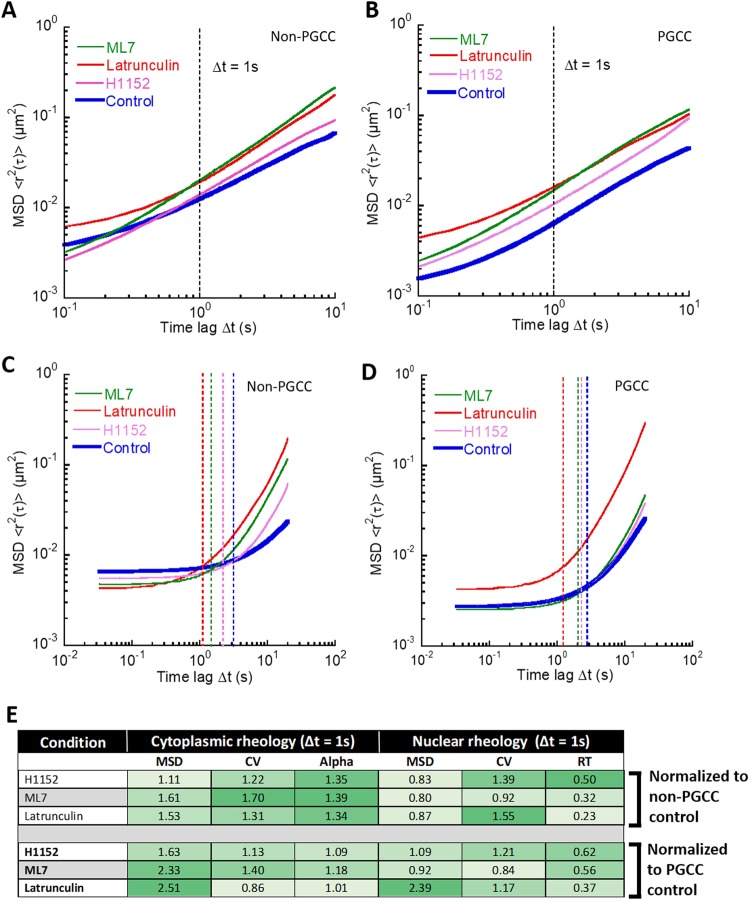
Biophysical phenotype of PGCCs is driven by actin cytoskeletal organization and mediated via the RhoA-ROCK1 pathway. (**A**) Total averaged MSD ensembles of particles embedded in non-PGCCs and (**B**) PGCCs tracked after 2-hour pretreatment with ML7 (MLCK inhibitor), H1152 (ROCK inhibitor) and latrunculin A (Actin cytoskeleton disruptor). (**C**) Total averaged MSD ensembles of chromatin granule motion in non-PGCCs and (**D**) PGCCs tracked after 2-hour pretreatment with ML7, H1152 and latrunculin A. (**E**) Summary table of inhibitor studies detailing MSD (at 1 second time lag), CV (coefficient of variation) and alpha (logarithmic slope of MSD as a function of time, for cytoplasmic mechanics) and RT (relaxation time, for nuclear mechanics). All results are normalized to either non-PGCC or PGCC untreated counterparts. Experiments were performed in triplicate.

**Figure 7:**
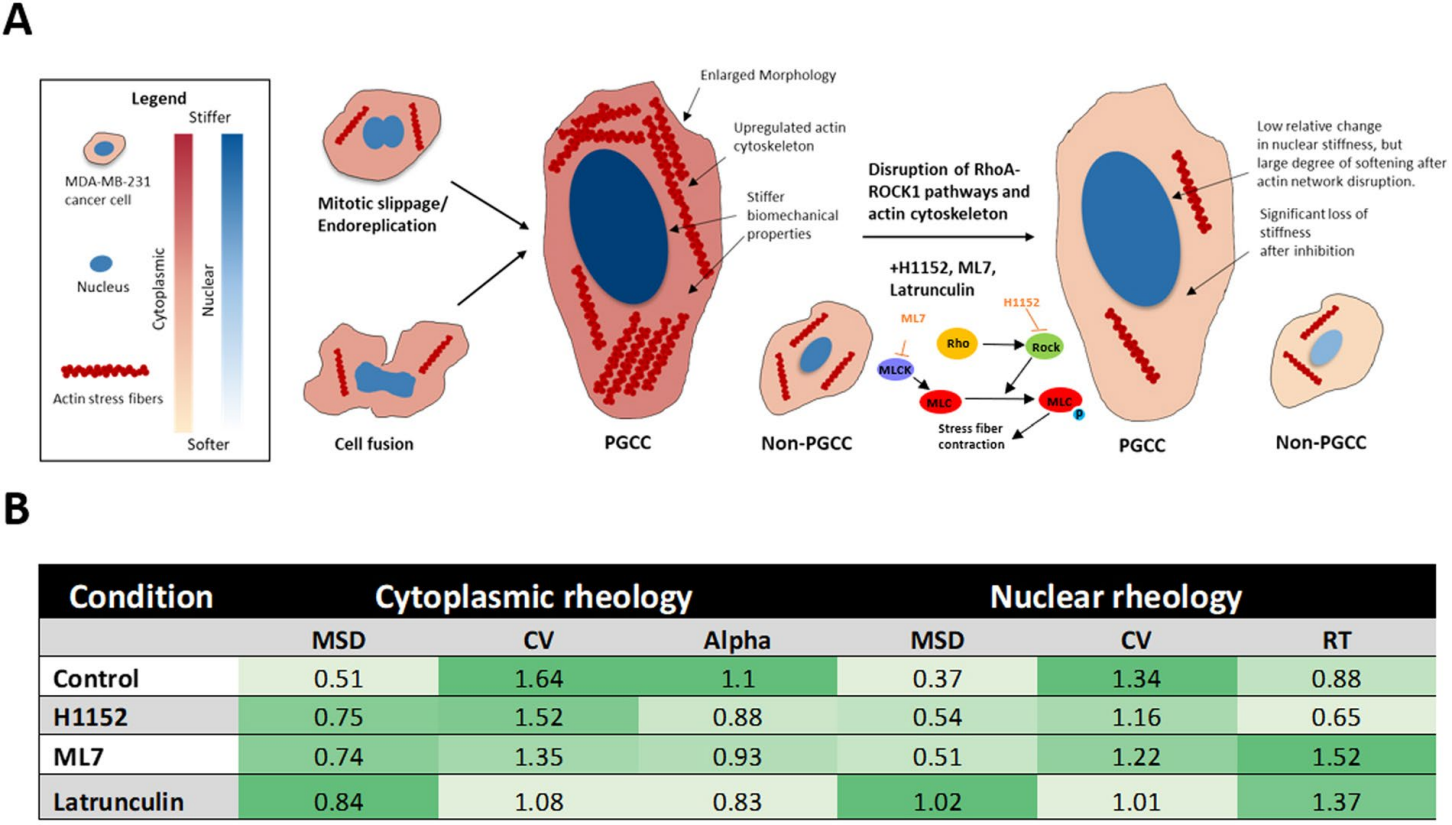
Mechanism driving differential biomechanics. (**A**) Schematic depicting formation of PGCCs and underlying cytoskeletal features that drive their stiffness. Upregulated actin stress bundles increase overall cell stiffness in PGCCs. RhoA-ROCK1 and actin network disruption leads to significant loss of cell stiffness. However, nuclear mechanics are less responsive to RhoA-ROCK1 inhibition but stiffness is fully restored to non-PGCC levels after actin disruption. (**B**) Summary table of inhibitor studies of PGCCs normalized to non-PGCCs, highlighting increased cytoplasmic sensitivity to inhibitors and nuclear sensitivity to actin disruption.

The underlying actin cytoskeleton of cells not only provide structural support but also defines the mechanical properties of the cell. A key regulator of the organization and dynamics of the actin network is the RhoA-ROCK1 mediated pathway^24^. Given the differential upregulation of actin stress bundle size and increased stiffness of PGCCs, involvement of the RhoA-ROCK1 and downstream targets is likely. By using latrunculin (actin cytoskeleton inhibitor), H1152 (ROCK inhibitor) and ML7 (MLCK inhibitor), we disrupted overall actin organization and dynamics of MDA-MB-231 cells. Again, particle and chromatin granule tracking was used to probe the biomechanical properties of PGCCs and non-PGCCs under treatment. When we treated PGCCs and non-PGCCs with latrunculin, H1152 and ML7, we observed no significant change in the slope of the ensemble averaged MSDs, but a reduction in amplitude, representing overall cytoplasmic softening in both PGCCs and non-PGCCs (Fig. 6A,B), as expected and in agreement with previous studies^25^. When we examined the nuclear mechanical properties after treatment with the inhibitors, non-PGCCs show a slight decrease in amplitude, as well as a decrease in relaxation time (Fig. 6C). In contrast, PGCCs responded significantly only for latrunculin treatment by increasing their amplitude and decreasing relaxation time, but had little response to H1152 and ML7 (Fig. 6D). When we examined the degree of cytoplasmic softening (as measured by a reduction in amplitude of ensemble MSDs at 1 second time lag normalized to respective controls), the effect of the inhibitors was much greater for PGCCs and increased compliance to levels similar to non-PGCCs (Figs 6E, 7B). This suggests PGCCs obtain their cytoplasmic biophysical phenotype via dysregulation in the RhoA-ROCK1 pathway and maintain it through upregulation of the actin cytoskeletal network. After this upregulation was disrupted, the PGCCs lost the underlying mechanisms that drove their stiffness, resulting in softening to levels closer to non-PGCCs (Fig. 7B). The same trend can be seen in the heterogeneity within these cells. After latrunculin inhibition, coefficients of variation of PGCCs normalized to non-PGCC cells reduced from 1.64-fold down to 1.08 (Fig. 7B), as the actin cytoskeletal elements causing localized regions of stiffness was disrupted. Similar to the cytoplasmic results, this effect of latrunculin on nuclear mechanics was more pronounced in PGCCs, rescuing nuclear compliance to levels close to non-PGCCs (Figs 6E, 7B), which suggests involvement of the actin network in maintaining nuclear stiffness, perhaps in the form of the perinuclear actin cap (Fig. S4). However, in contrast to the cytoplasmic results, the PGCCs had a significantly weaker response in terms of a reduction in relaxation time and MSD amplitude when treated with H1152 and ML7 compared to non-PGCCs (Fig. 6E), pointing towards a disruption in the RhoA-ROCK1 pathway mediated coupling of cytoskeletal and nuclear mechanics.

## Discussion

It has been long established that cancer relapse is often triggered by a drug-resistant subpopulation as opposed to tumor-wide chemoresistance^26^. Cancer PGCCs have previously been observed both *in vivo* and in culture and are especially prominent in high-grade, late-stage and treatment resistant cancers^15,27^. However, these giant cancer cells are often overlooked due to the low population and inability to replicate normally, often attributed to senescence^28^. In this study we presented data demonstrating the relevance and importance of these PGCCs in cancer, consistent with mounting research which allude to the critical role these PGCCs play as the elusive treatment-resistant subpopulation responsible for relapse. We show that this small but significant subpopulation exists in the MDA-MB-231 TNBC cell line and is amplified upon PTX treatment, even at low dosages (Fig. 1A,C,F). To confirm PGCCs have an inherent survival advantage, we observed MDA-MB-231 cells under treatment. Time-lapse microscopy revealed that the PGCCs were less responsive to PTX in comparison non-PGCCs. This suggests the unique phenotype expressed by these large cells conferred a survival advantage to PTX treatment, perhaps in part due to their dormancy or abnormal replication cycle, which corroborates with previous studies where PGCCs observed in prostate cancer had apparent resistance to docetaxel^29,30^. Due to the preferential targeting of cells undergoing mitosis by PTX, these PGCCs can evade treatment due to their slow proliferation and unconventional mechanisms of division, which have been described previously^31,32^.

Although the exact mechanism by which PGCCs are formed is not known, previous studies have documented their formation as the result of endoreplication or cell fusion, and most likely a combination of both^33,34^. The presence of a large, broad peak seen in DNA content analysis (Fig. 1B) suggests a multitude of mechanisms contribute to increased ploidy. If the underlying mechanism was solely endoreplication or mitotic slippage, we would expect to see a sharp, distinct peak at 4 or 8N^35–37^. Instead, a combination of the two or more processes (such as cell fusion) must be occurring in order to generate a spectrum of polyploidy that we observed. Given the diverse nature of their formation, it is not surprising that the heterogeneity of this PGCC subpopulation is greatly increased compared to non-PGCCs. Firstly, both the cytoplasmic and nuclear sizes of PGCCs span an extensive range, much larger than diploid MDA-MB-231 cells in breadth. Second, MPT analysis show increased heterogeneity in the biophysical properties of both cytoplasmic and nuclear mechanics, as evidenced by the increased coefficient of variation (Figs 2C, 3C). To put this finding in context, cancer heterogeneity has been firmly associated with metastatic dissemination and invasion, and can be used as a prognostic marker for disease progression^38,39^. The highly diverse PGCCs not only represent an extremely heterogeneous population, but also serves to raise the overall heterogeneity of the tumor. With the increased prominence of these cells in tumors after treatment in turn leading to higher heterogeneity, it is clear the relevance these cells bear on cancer aggression and relapse potential.

Inhibitor studies and morphological characterization reveals inherent dysregulations of PGCCs compared to non-PGCCs in their morphology, cytoskeletal organization and mechanics. Surprisingly, we noted similarities in the biophysical properties of PGCCs and stromal cells. Overall, these polyploidal cells display a distinctive mode of migration driven by mechanisms unlike regular cancer cells. Previous work demonstrated cytoplasmic stiffness is correlated with an increased migratory phenotype in fibroblasts^40^. In contrast, the mechanical phenotype of cancer cells has been associated with lower stiffness compared to stromal counterparts, and cell stiffness is inversely correlated with an invasive migratory phenotype^41,42^. The increased persistence in migration (Fig. 4E) and cytoplasmic stiffness observed in PGCCs (Fig. 2A,B) is a trend comparable to migratory fibroblasts but not cancer cells, despite its origins as a highly invasive TNBC. Furthermore, qualitative observation of these PGCCs in culture and during migration show a close physical interaction between the large cells and surrounding cancer cells, akin to the support stromal monolayer used to promote cell proliferation in culture^43^. Non-polyploidal MDA-MB-231 cells can often be observed clustering and seemingly attached to the sides or even on top of PGCCs (Fig. S5). This suggests an increased expression of cell-cell adhesion proteins on their surface, allowing them to adopt a stromal-like role in the context of the tumor microenvironment. Collectively, these unique stromal-like features of the PGCC phenotype in addition to their enlarged morphology reveal an underlying dysregulation in the pathways that control cellular adhesion and overall cytoskeletal organization.

We examined the cytoskeletal network of the PGCCs to gain insight on the underlying mechanisms of their unique biophysical phenotype. Interestingly, PGCCs did not possess the typical polarity phenotype, normally driven by the microtubule organizing center (MTOC) and actin dynamics^44^. PGCCs had an apparent loss of polarity, due to their ploidy and apparent presence of multiple MTOCs (Figs 2D, S1F). Despite the absence of polarity, PGCCs were surprisingly motile. Likely, their persistent motility is in part due to a dysregulation in traditional mechanisms of polarization^45^ and their abnormal actin structure and turnover might lead to decreased ability to alter direction, resulting in increased persistence. Regulation of cytoplasmic actin plays a crucial role in cell movement and cancer metastasis^46^. Biophysical characterization of cells and their actin organization show a direct correlation between stress fiber formation and mechanical stiffening^47,48^. Quantification of actin stress fibers in PGCCs show both increased fiber thickness and length (Fig. 3), and demonstrates their abnormal upregulation of actin cytoskeletal elements, manifesting in stiffer rheological properties and increased migratory persistence. Increased sensitivity to RhoA-ROCK1 inhibitors points towards the involvement of a dysregulated pathway and overall actin cytoskeletal network as the underlying mechanism for the altered cytoplasmic biophysical properties of PGCCs (Fig. 7A). This finding is important in the context of cancer progression, as the dysregulation of the RhoA-ROCK1 pathway in cancer and its link to increased malignancy has been well documented^49,50^. Furthermore, decreased response to RhoA-ROCK1 inhibition in PGCC nuclear mechanics points towards an uncoupling of nuclear and cytoplasmic mechanics. Nonetheless, involvement of actin cytoskeleton in PGCC nuclear mechanics cannot be fully discounted, as actin disruption with latrunculin preferentially softened PGCC nuclei compared to non-PGCCs, increasing compliance to non-PGCC levels. This demonstrates involvement of actin structures in regulating the inherent stiffness of PGCC nuclei, possibly through the actin perinuclear cap. The perinuclear actin cap is comprised of thick bundled actin filaments spanning the apical surface of cell nuclei (Fig. S4). Previous research have shown involvement of the actin cap in regulating nuclear mechanotransduction and shape^18^. Immunostaining of PGCCs revealed a distinct perinuclear cap (Fig. S4B), a feature that is normally lost or diminished in disease states such as laminopathies and invasive cancer. The unusual presence of this actin cytoskeletal feature explains the abnormal stiffness observed in PGCCs, in addition to the dynamic ability of PGCCs to alter their nuclear shape during directional migration.

In sum, our studies serve to elucidate the stiffer biophysical properties of PGCCs and highlight the importance of this previously overlooked subpopulation, which is linked to cancer progression, treatment resistance and relapse. We elucidated their chemoresistant phenotype, most likely due to dormancy in addition to abnormal mechanisms of cell division as a potential method of treatment evasion. Furthermore, PGCCs appear to contain stromal-like biomechanical properties which can prime the local tumor microenvironment for cancer development and progression. Lastly, these PGCCs exhibit increased levels of heterogeneity and dysregulation, two characteristics highly correlated with cancer metastasis and aggression. With the increasing incidence rate of these cells in more aggressive tumors or after treatment with chemotherapeutics, their role in dictating overall cancer progression becomes extremely important in the context of treatment and disease survival. Targeted therapeutic approaches to eliminate PGCCs could include low-dose inhibition of RhoA-ROCK1 adjuvant, mitigating the biomechanical phenotype that permits their survival in the face of mechanical stresses, potentially reducing relapse potential and improving patient prognosis.

## Methods

### Cell Culture

MDA-MB-231 human triple negative breast cancer cells (ATCC) were cultured in DMEM/Ham’s F12 50/50 mix (Corning) supplemented with 10% FBS (Atlanta Biologicals) and 1% penicillin-streptomycin (Corning). Cells were maintained at 37 °C and 5% CO_2_.

### Immunofluorescence staining and analysis

MDA-MB-231 cells cultured on glass coverslips were stained for actin and microtubule cytoskeletal networks as described previously^51^. A Nikon Eclipse Ti inverted fluorescent microscope equipped with a 60x oil immersion lens was used to image the coverslips. CurveAlign(LOCI) MATLAB software was used to quantify actin stress fiber structures. DAPI stained nuclear images were acquired through a 20x Nikon objective. The collected images were then batch processed through CellProfiler (Broad Institute) to obtain nuclear size and shape factor. For DAPI quantification of PGCCs, we used a definition of a nuclear area at least 2.5x the average nuclear size of the parent population. This cutoff excludes cells in the G2/M phase undergoing normal mitosis. We have also conducted flow cytometry based DNA cell cycle analysis on the same population to confirm the PGCC population, and quantification yielded results matching closely with our imaging results using the 2.5x cutoff (Fig. 1B,C). For MDA-MB-231s, this cutoff was around 250 µm^2^.

### Motility experiments

24 well plates were coated with 20 μg/mL type I rat tail collagen (Corning) for 1 hour prior to adding cells. MDA-MB-231 cells were seeded at a density of 5000 cells per well and allowed to adhere for 24hrs prior to being stained with Hoechst 33342 (1 μg/mL). Cells were tracked every 10 minutes for 12 hours on a Nikon Eclipse Ti inverted fluorescent microscope equipped with an environmental chamber (37 °C and 5% CO_2_). Image stacks were processed in a custom MATLAB algorithm to track the x-y coordinates of cell nuclei to determine cell velocity.

### Scratch assay

Cells were grown to confluence on a glass-bottom 35 mm petri dish (Matek). A 10 μL pipette tip was used to create an even gap in the confluent monolayer. The dishes were then washed with PBS to remove detached cells and subsequently stained with Hoechst 3342 (1 μg/mL). Cells were placed on Nikon inverted microscope and maintained at 37 °C and 5% carbon dioxide, while being imaged for 12 hours at 60-minute intervals.

### Flow cytometry cell cycle analysis

MDA-MB-231 cells were trypsinized and fixed in ice cold 70% ethanol and stored at −20 °C overnight. The cells were then washed with PBS and incubated (3 hours at 4 °C) in 1 mL 3.8 mM sodium citrate (Fisher chemicals), 50 μg/mL RNAse A and 50 μg/mL PI solution. Flow cytometry analysis was done on an easyCyte HT (Guava instruments) flow cytometer.

### Multiple particle tracking analysis

Intracellular rheology was probed via analyzing the thermal motions of injected fluorescent nanoparticles as previously described^52–54^. Briefly, 200-nm fluorescent carboxylate particles were ballistically injected into cell cytoplasm using a PDS-1000 Biolistic Helium Particle Injection System (BioRad). A Nikon Eclipse Ti inverted fluorescent microscope equipped with a Nikon CFI Apochromat TIRF 100x oil-immersion lens (NA = 1.49) was used to capture high spatial-temporal resolution videos of particle movement within the cell. Particle movements were tracked in a custom MATLAB algorithm as described previously^55,56^, using a band-pass filter to isolate particles and a Hungarian linker algorithm to construct tracks of the particles based on their intensity weighted centroid. Ensemble averaged time dependent particle mean square displacements (MSDs) were calculated and reported along with the logarithmic slope of the MSD (α). Nuclear particle tracking was done in similar fashion, except chromatin granules within the nucleus were tracked instead of injected particles. Ensemble averaged time dependent particle mean square displacements (MSDs) were calculated and reported along with the relaxation time (when the logarithmic slope of the MSD reaches 0.5).

### Inhibitor studies

H1152 (Enzo Life Sciences, lot: 03301611), ML7 (Enzo life sciences, lot: 0391631), and latrunculin (Cayman chemicals, 0506867-2)were used to inhibit components of RhoA-ROCK1 mediated signaling and overall actin cytoskeletal network at concentrations of 1 μM, 10 μM and 0.4 μM, respectively as described previously^51^. Cells were treated for 1 hour prior to motility and multiple particle analysis, and 24 hours prior to imaging. Taxol concentrations of 25, 50 and 100 nM were used to study PGCC formation over the course of 48 hours. PGCC and non PGCC response to high concentration Taxol was conducted at 100 nM for 24 hours, while being imaged for 24 hours at 30-minute intervals.

### Statistics

All experiments were done with 3 biological replicates and minimum 3 technical replicates, with at least 15 cells unless otherwise indicated. Student’s t-test was used to compare two conditions. A p-value of less than 0.05 was considered significant (*p < 0.05, **p < 0.01, ***p < 0.001). Data is represented as the mean ± standard error of the mean for at least 3 experiments.

## Electronic supplementary material

Supplemental Data

Supplementary video 1
