# Supplementary material for: Dysregulation in Actin Cytoskeletal Organization Drives Increased Stiffness and Migratory Persistence in Polyploidal Giant Cancer Cells
Source: Sci Rep. 2018 Aug 9;8:11935. doi: 10.1038/s41598-018-29817-5 (PMC6085392; doi:10.1038/s41598-018-29817-5)
Supplement: Supplementary file 1 — Supplemental Data [file 41598_2018_29817_MOESM1_ESM.docx]

Dysregulation in Actin Cytoskeletal Organization Drives Increased Stiffness and Migratory Persistence in Polyploidal Giant Cancer Cells

Botai Xuan^1^, Deepraj Ghosh^1^, Emily Cheney^1^, Elizabeth Clifton^1^, Michelle R. Dawson^1,2,3*^

^1^Brown University, Department of Molecular Pharmacology, Physiology, and Biotechnology, Providence, 02912, USA

^2^Brown University, Center for Biomedical Engineering, Providence, 02912, USA

^3^Brown University, School of Engineering, Providence, 02912, USA

^*^Corresponding. michelle_dawson@brown.edu


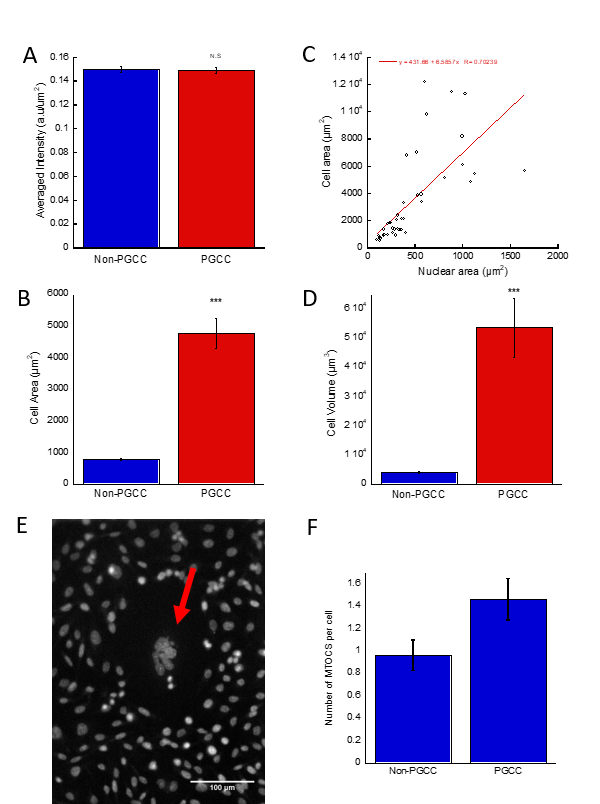


**Figure S1: Supplemental morphological analysis.**

**(A)** Fluorescence intensity as a function of area for PGCC and non-PGCC nuclei stained with DAPI. **(B)** Nuclear and cell area correlation for PGCC and non-PGCCs. **(C)** Average cell size and **(D)** volume analysis of PGCC and non-PGCCs show increased differences in volume compared to area, suggested differences in thickness not reflected in a 2-D projection. **(E)** DAPI-stained nuclei of PGCC (red arrow) surrounded by non-PGCCs, highlighting key differences in nuclear size and shape. **(F)** Quantification of MTOCs in tubulin stained non-PGCC and PGCCs revealed higher average number of MTOCs in PGCCs compared to non-PGCCs.

All experiments were performed in triplicate (unless noted otherwise). Results are reported as the mean± SEM. Significance is indicated as **p<0.05*, ***p<0.01*,****p<0.001*


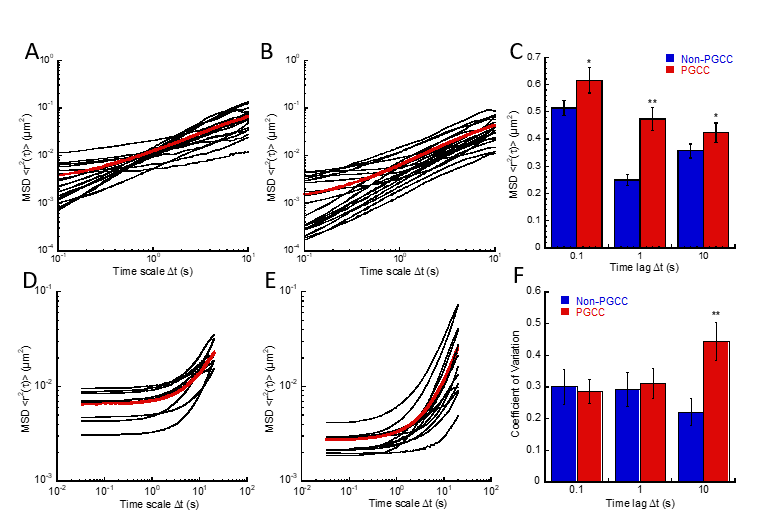


**Figure S2: Cytoplasmic and nuclear analysis averaged for each cell.**

**(A)** Ensemble averaged MSDs for each cell derived from particle motion embedded within cell cytoplasm of non-PGCCs and **(B)** PGCCs show decreased particle motion and increased stiffness in PGCCs (100+ traces, 30+ cells). **(C)** Coefficient of variation analysis for ensemble MSDs of each cell of non-PGCC and PGCCs reveal increased heterogeneity in PGCCs. **(D)** Ensemble averaged MSDs for each cell derived from chromatin granules within the nucleus of non-PGCCs and **(E)** PGCCs show decreased particle motion and increased nuclear stiffness in PGCCs (100+ traces, 30+ cells). **(F)** Coefficient of variation analysis for chromatin granule ensemble MSDs averaged for each cell of non-PGCC and PGCCs reveal increased heterogeneity in PGCCs. All experiments were performed in triplicate (unless noted otherwise). Results are reported as the mean± SEM. Significance is indicated as **p<0.05*, ***p<0.01*,****p<0.001*


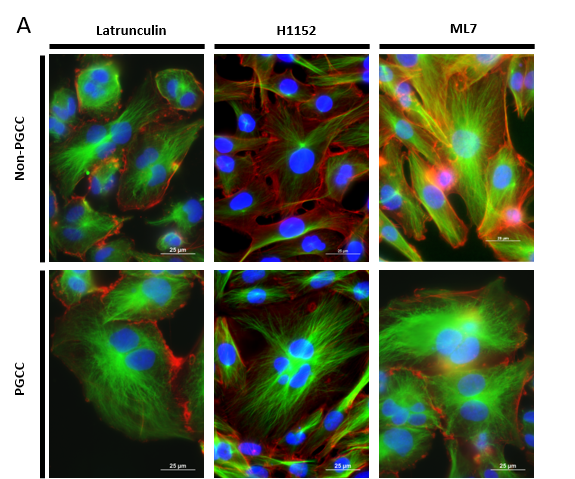


**Figure S3: Fluorescent images of cells after inhibitor treatment.**

**(A)** Fluorescent images of non-PGCC and PGCCs treated with H1152, ML7 and latrunculin for 48 hours then stained with Phalloidin (F-actin, *red*), Anti-α-tubulin (Microtubule, *green*), and DAPI (Nucleus, *blue*).


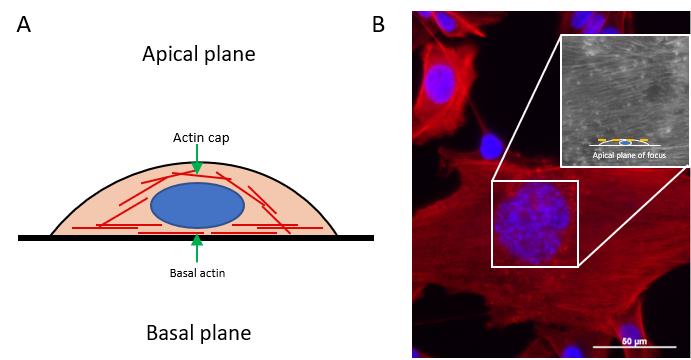


**Figure S4: Presence of a perinuclear actin cap in PGCCs.**

**(A)** Schematic describing the nature of a perinuclear actin cap. **(B)** Fluorescent image of non- a PGCC stained with Phalloidin (F-actin, *red* and DAPI (Nucleus, *blue*).


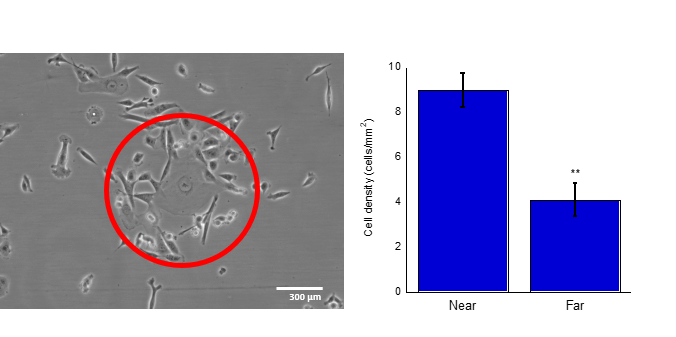


**Figure S5: Clustering of non-PGCCs around PGCCs**

**(A)** Non-PGCCs clustering near the surface of PGCCs. **(B)** Quantification of cell density near PGCCs or more than 1 cell length away from PGCCs (n=3)

**Supplementary video 1: 16-h time lapse of MDA-MB-231 cells during random motility experiment**
